# Supplementary material for: A street-view-based method to detect urban growth and decline: A case study of Midtown in Detroit, Michigan, USA
Source: PLoS One. 2022 Feb 8;17(2):e0263775. doi: 10.1371/journal.pone.0263775 (PMC8824339; doi:10.1371/journal.pone.0263775)
Supplement: Attachment — Submitted filename: PONE-D-21-18677_R2_responses to reviewers.docx [file pone.0263775.s002.docx]

Manuscript Number: PONE-D-21-18677

Responses to the reviewers’ comments

“A street-view-based method to detect urban growth and decline: A case study of Midtown in Detroit, Michigan, USA”

**Reviewer 1**

*General comments:*

Reviewer #1: The author has addressed all the comments I made in the previous version. And I think this paper is qualified enough to be published.

>> **Response & Correction**

We would like to thank the reviewer 1 for reviewing our manuscript and giving us valuable suggestion and comments. With your comments and suggestion, we improved our manuscript. Thank you very much.

**Reviewer 2**

*General comments:*

Reviewer #2: By the last round of revision, the authors have addressed my concerns. Then I would like to recommend the editor broad to accept this paper. Before publishing, the author should improve again the writing quality of this manuscript, and especially focus on the introduction part and state more clearly about the research motivation and contributions.

>> **Response & Correction**

We would like to thank the reviewer 2 for supporting our revision and suggesting the improvement. According to your suggestion, we have carefully revised the manuscript. In particular, we have added motivation and contribution in the section of Introduction. It reads:

*“… In particular, yearly changes in street view images motivate this study to focus on urban changes at a street level. This novel method to analyze urban changes at a street level seeks to contribute to automation and efficiency in identifying growth and declines in urban areas. ….”*
